
JOURNAL INFORMATION
==============================
NLM Title Abbreviation: Buffalo Med J
NLM Journal ID: 0027534

Buffalo Medical Journal

ISSN: 1040-3817
Publisher: Biomedical Journal Digitization Project

ARTICLE INFORMATION
==============================
PMCID: PMC8726915
PMID: 36885295
Article version: 1
Subjects: Miscellany

Miscellany

Print publication date: 1909 May
Volume: 64
Issue: 10
First page: 584
Last page: 584
License: This work is free of known copyright restrictions. For more information, please see PMC Back Issue Digitization.
License URL: https://creativecommons.org/publicdomain/mark/1.0/

==============================

extra cloth. Price $4.00 net. F. A. Davis Company, publishers, 1914-16 Cherry Street, Philadelphia, Pa.

Annual Report of the Surgeon General of the Public Health and Marine Hospital Service of the United States, giving the operations of the service, fourth fiscal year, 1908. Washington Government Printing Office. 1909.

Mortality Statistics, Department of Commerce and Labor, Bureau of the Census, 1907 Eighth Annual Report, by S. N. D. North, Director. Washington: Government Printing Office. 1909.

Atlas and Epitome of External Diseases of the Eye. By Professor Dr. O. Haab, of Zurich. Edited, with additions, by George E. deSchweinitz, M.D., Professor of Ophthalmology, University of Pennsylvania. Third revised edition. With 101 colored lithographic illustrations on 46 plates and 244 pages of text. Philadelphia and London: W. B. Saunders Company, 1909. Cloth, $3.00 net.

Atlas and Epitome of Ophthalmoscopy and Ophthalmoscopic Diagnosis. By Professor Dr. O. Haab, of Zurich. Edited, with additions, by George E. deSchweinitz, M.D., Professor of Ophthalmology, University of Pennsylvania. Second revised edition. With 152 colored lithographic illustrations and 94 pages of text. Philadelphia and London: W. B. Saunders Company, 1909. $3.00 net.

---

### MISCELLANY.

Army Medical Corps Examinations.—The Surgeon General of the Army announces that preliminary examinations for appointment of first lieutenants in the Medical Corps of the Army will be held on July 12, 1909, at points to be hereafter designated.

Full information concerning the examination can be procured upon application to the "Surgeon General, U. S. Army, Washington, D. C."

---

Medical Interne.—Government Hospital for the Insane. June 16, 1909. The United States Civil Service Commission announces an examination on June 16, 1909, at the usual places, to secure eligibles from which to make certification to fill at least two vacancies in the position of medical interne (male), Government Hospital for the Insane, Washington, D. C., at $600 per annum each, with maintenance, and vacancies requiring similar qualifications as they may occur in that hospital.

FREE SAMPLE of a new Patent Two Finger Obstetrical Examination Cot will be sent to Physicians sending card or prescription blank. Other Novelties. Address, Medical Equipment Co., 149 West 23d. Street, New York.

WANTED slightly used instruments and all kinds of office equipment in good condition. Fair prices for reliable goods. Distance no object. Write Henderson, 149 West 23d. Street, New York.
